# A study on electrospray mass spectrometry of fullerenol C_60_(OH)_24_

**DOI:** 10.3762/bjoc.9.145

**Published:** 2013-07-02

**Authors:** Mihaela Silion, Andrei Dascalu, Mariana Pinteala, Bogdan C Simionescu, Cezar Ungurenasu

**Affiliations:** 1 Petru Poni Institute of Macromolecular Chemistry, Centre of Advanced Research in Bionanoconjugates and Biopolymers, Aleea Grigore Ghica Voda 41A, Iasi 700487, Romania; 2 Department of Natural and Synthetic Polymers, “Gheorghe Asachi” Technical University of Iasi, 700050 Iasi, Romania

**Keywords:** electrospray, fullerenol C_60_(OH)_24_, mass spectrometry

## Abstract

Full characterization of fullerenol C_60_(OH)_24_ by HPLC ESI-MS in negative and positive ionization modes was achieved. Fragmentor voltage and capillary voltage were optimized in order to obtain a good signal stability and the best peak intensity distribution for the fullerenol C_60_(OH)_24_ in both negative and positive modes. While the predominant base peak observed for C_60_(OH)_24_ in the negative ionization mode was [M − H]^−^ at *m*/*z* 1127, those observed in the positive mode were multiply charged [M − H_2_O + 4H]^4+^ at *m*/*z* 279 and [M − 12H_2_O + 2NH_3_ + 6H]^6+^ at *m*/*z* 158.

## Introduction

Because of their potential for chemical tunability and exciting range of biological activities as glutamate-receptor antagonists [1] and antiproliferative [2–3], neuroprotective [4–7], and anticancer agents [8–13], polyhydroxylated [C_60_]fullerenes, C_60_(OH)_x_, have received much attention in recent years. However, to the best of our knowledge, except for the compositionally and structurally well characterized C_60_(OH)_24_, prepared by alkaline hydrolysis of C_60_Br_24_ [14–15], most of these fullerenols are not pure C_60_(OH)_x_, but a complex mixture of products with an average composition of C_60_(OH)_x–y_, C_60_O_x_(OH)_y_ [16–19] or C_60_(OH)_x_(ONa)_y_ [20].

Therefore, the HPLC separation and accurate measurement of the molecular weight for structure characterization by electrospray ionization mass spectrometry (ESI-MS) have become essential for fullerenol research. Fullerenols C_60_(OH)_18–44_ are very small neutral molecules with the highest density of hydroxy groups on a given particle surface (up to 10.7 OH/nm^2^) [21] achieved so far, complicating spectra acquisition if the correct conditions are not used. Initial attempts made by Isaacson et al [22] in 1994 to develop mass-spectrometric methods for fullerenols using ESI-MS with commercially available C_60_(OH)_24_ and C_60_(OH)_22–24_ dissolved in acidic, basic, and neutral solutions did not produced ions diagnostic of C_60_(OH)_x_. Afterwards, very few isolated attempts have been made on the identification of fullerenols [23] and/or their derivatives [24–28] by ESI-MS analysis, and very little detail was provided. We carried out an experimental investigation on ESI-MS of fullerenol C_60_(OH)_24_ over a wide range of total capillary and fragmentor voltages.

Herein, we are happy to report the first experimental evidences that fullerenols are ESI-MS-inactive compounds in aqueous media at low and medium capillary and fragmentor voltage under negative ionization conditions, but they are ESI-MS-active compounds in aqueous and ammonia media, in both negative and positive ionization mode at a capillary voltage of 4.5 kV and a fragmentor voltage of 400 V with no C_60_-cage fragmentation.

## Results and Discussion

### Nomenclature and ionization mechanisms

**Scheme 1:**
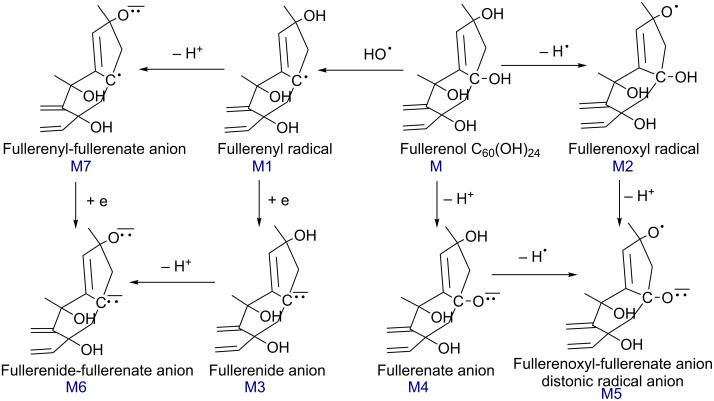
Proposed mechanisms for the formation of fullerenol anions and distonic radical anions observed by (−)ESI-MS spectra of C_60_(OH)_24_ in pure water.

In the discussion, we will use the expressions protonated [M + H]^+^ and deprotonated [M − H]^−^ molecules, and distonic deprotonated molecules for those identified from a deprotonated molecule plus loss of HO**^•^** and/or H**^•^** radicals [M − HO^•^ − H^•^ − H]^−^. However, while only radical-ion pairs have been identified by ESI-MS as distonic ions, the distonic deprotonated molecules observed in (−)ESI-MS spectra of C_60_(OH)_24_ contain a different number of charges and radicals on the C_60_ cage. Consequently, these distonic species should be described as quasi-distonic radical anions. The proposed mechanisms for the formation of fullerenol C_60_(OH)_24_ anions and distonic radical anions are shown in Scheme 1.

For naming these individual radical species using “yl” and “oxyl” suffixes the terms fullerenyl for radicals generated by the loss of HO**^•^** (M1 in Scheme 1) and fullerenoxyl for radicals generated by the loss of H^•^ (M2 in Scheme 1) will be used (IUPAC 2002) [29]. Radical anions formed by electron addition to a fullerenyl radical shall be described as fullerene carbanions and referred to as fullerenide anions (M3 in Scheme 1) and [mM + ne]^m–n∙^ shall be described as molecular anions. As regards to the observation of distonic fullerenyl-fullerenate M7 ions, such distonic dehydroalcoxide radical anions, namely dehydrophenoxide radical anions, were first observed by Bowie et al [30] and recently investigated by ESI-MS by Mariappandar et al [31].

Anions formed by the deprotonation of OH (M4 in Scheme 1) shall be named as fullerenate anions. Consequently, the ions M6 shall be named fullerenide-fullerenate anions, and ions M5 and M7 shall be named fullerenoxyl-fullerenate and fullerenyl-fullerenate distonic anions, respectively. To avoid any confusion with dissociative electron attachment (DEA) (addition of a free electron to a gas-phase molecule to give an anion and a neutral molecule via a superexcited anion), the term quasi-electron-capture will be used for the addition of an electron to a fullerenyl radical M7 to generate a carbanion M6.

### Negative-ionization-mode mass spectra

The negative-ion ESI-MS spectra and ionization data of C_60_(OH)_24_ in ultrapure water and in the presence of small amounts of aqueous ammonia will be discussed here.

#### Fullerenol in pure water

**Figure 1:**
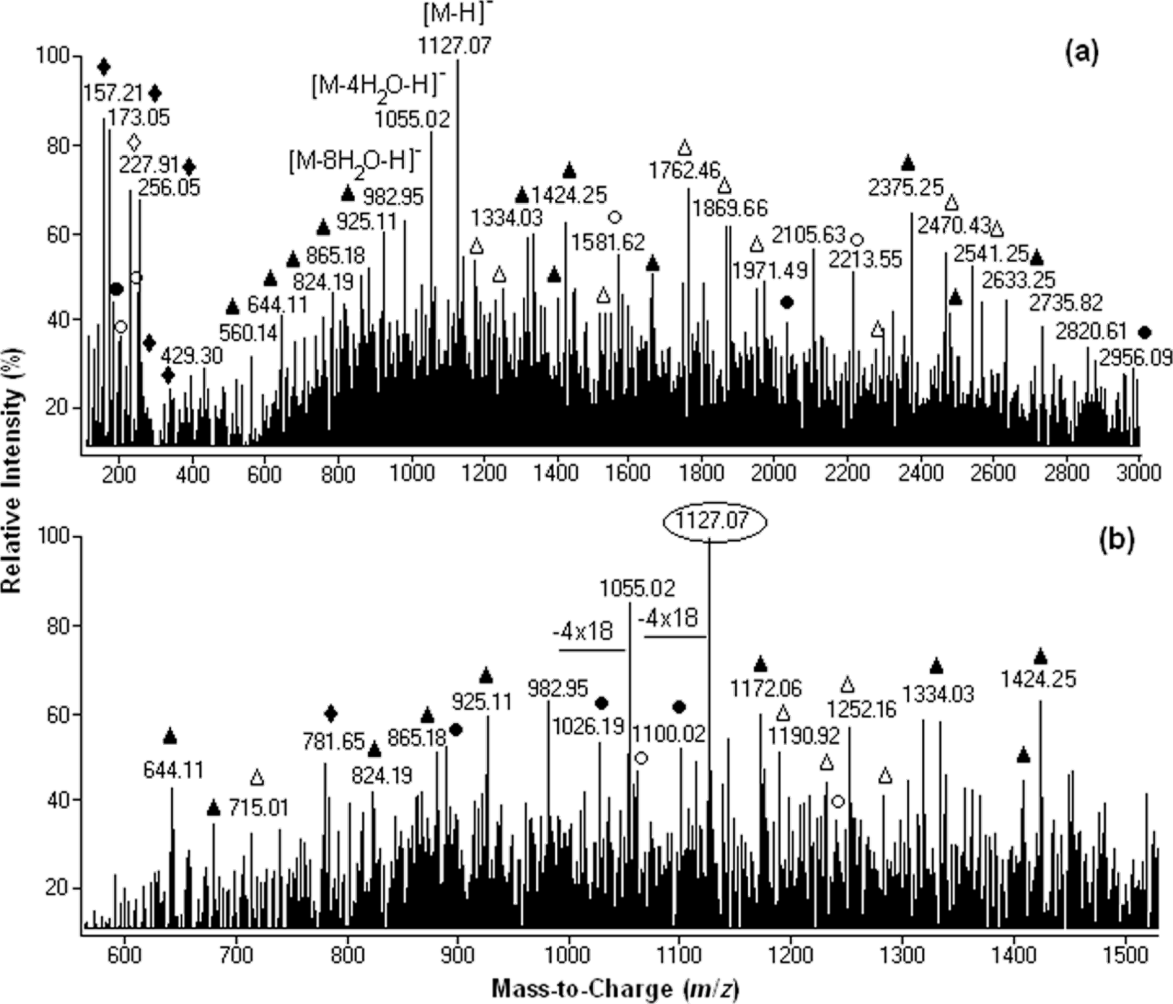
Negative-ion mass spectra for a 0.5 × 10^−5^ M solution of C_60_(OH)_24_ in ultrapure water: (a) full scan spectrum (b) *m*/*z* range 600–1500. (●[M(n) − *n*H_2_O − xH]^x-^, ▲[M(n) + *n*H_2_O(m) − xH]^x−^, ♦[M(n) − H_2_O(m) − yHO^•^ + ye − xH]^(x+y)−^, ○[M(n) − *m*H_2_O − yH^•^ − xH]^x−y•−^, ∆[M(n) + H_2_O(m) − yH^•^ − xH]^x−y•−^, ◊[M − *m*H_2_O − yHO^•^ − zH^•^ − xH]^x−(y+z)•−^).

Three distinct regions can be identified in the mass spectra: *m*/*z* 100–600, *m*/*z* 600–1500 (Figure 1b) and above *m*/*z* 1500. Between *m*/*z* 600 and 1500, the most abundant ion is centered at *m*/*z* 1127 and corresponds to the singly charged base molecular ion [M − H]^−^ generated through the deprotonation of fullerenol C_60_(OH)_24_.

**Scheme 2:**
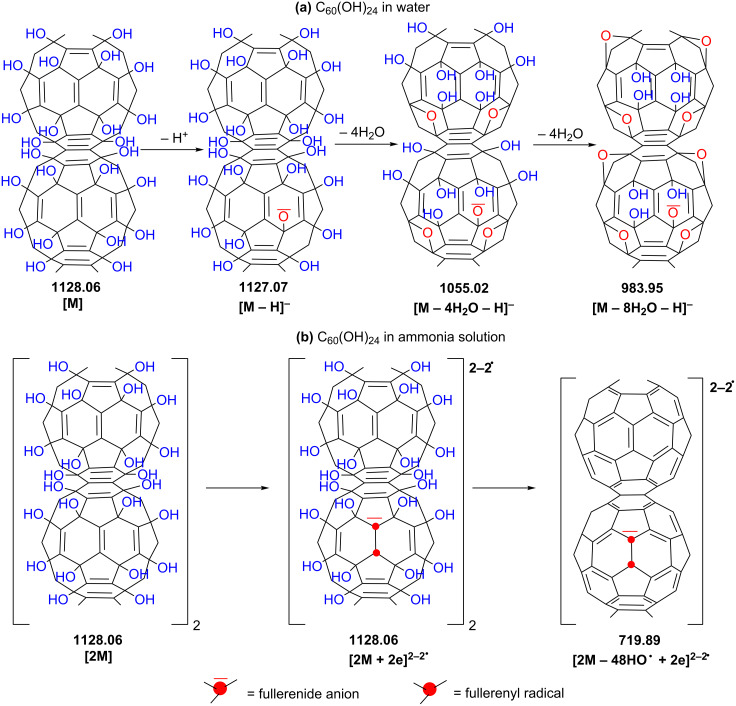
Examples of proposed structures for the main deprotonated molecules and final distonic molecular ion observed by (−)ESI-MS of C_60_(OH)_24_ in pure water (a) and aqueous ammonia solution (b).

As shown in Table 1 (entries 1–3) and Scheme 2, consecutive weight loss of 18 mass units from the deprotonated molecule [M − H]^−^ at *m*/*z* 1127 was clearly observed through the presence of singly charged ions centered at *m*/*z* 1055 [M − 4H_2_O − H]^−^ and *m*/*z* 983 [M − 8H_2_O − H]^−^.

**Table 1:** Summary of the dominant ESI-MS negative ionic species of fullerenol C_60_(OH)_24_ in ultrapure water (relative abundance, assignments, and *m*/*z* values) at 4.5 kV capillary voltage and 400 V fragmentor voltage.

Entry	Relative intensity (%)^a^	Assignments	*m/z*		

			Calculated	Found	Deviation

1	100	[M − 1H]^−^	1127.0570	1127.0719	−0.0149
2	83	[M − 4H_2_O − 1H]^−^	1055.0146	1055.0172	−0.0026
3	63	[M − 8H_2_O − 1H]^−^	982.9722	982.9495	0.0227
4	55	[M + 7H_2_O −1H**^•^** − 1H]^−1^**^•^**	1252.1234	1252.1617	−0.0383
5	54	[2M + 12H_2_O − 1H**^•^** − 1H]^−1^**^•^**	2470.2432	2470.4280	−0.1848
6	54	[3M − 12H_2_O − 3H**^•^** − 2H]^2−3^**^•^**	1581.5141	1581.6201	−0.1060
7	63	[3M + 20H_2_O − 3H**^•^** −2H]^2−3^**^•^**	1869.6852	1869.6562	0.0290
8	20	[M − 8H_2_O − 3OH**^• −^** 3H]^3−3^**^•^**	309.9961	309.9241	0.0720
9	51	[2M + 19H_2_O − 3H]^3−^	865.1027	865.1792	−0.0765
10	52	[3M + 18H_2_O − 12H**^•^** − 3H]^3−12^**^•^**	1231.0894	1231.0091	0.0803
11	49	[4M + 9H_2_O − 9H**^•^** − 3H]^3−9^**^•^**	1554.0869	1554.0754	0.0115
12	65	[6M + 20H_2_O − 3H]^3−^	2375.1924	2375.2462	−0.0538
13	71	[M − 4H_2_O − 4H**^•^** − 8HO**^•^** − 4H]^4−12^**^•^**	227.9846	227.9089	0.0757
14	55	[M − 1H**^•^** − 6H_2_O − 4H]^4−1^**^•^**	253.7405	253.7368	0.0037
15	70	[M − 2H_2_O − 4OH**^•^** + 4e]^4−^	256.0082	256.0519	−0.0437
16	32	[M + 62H_2_O − 4H]^4−^	560.1727	560.1382	0.0345
17	43	[2M + 26H_2_O − 4H]^4−^	680.0935	680.0391	0.0544
18	62	[4M + 10H_2_O − 4H]^4−^	1172.0835	1172.0648	0.0187
19	65	[4M + 66H_2_O − 4H]^4−^	1424.2319	1424.2480	−0.0161
20	71	[6M − 3H**^•^** + 16H_2_O − 4H]^4−3^**^•^**	1762.3759	1762.4616	−0.0857
21	55	[4M − 6H_2_O − 5H]^5−^	879.8313	879.8669	−0.0356
22	51	[5M − 2H**^•^** − 19H_2_O − 5H]^5−^**^•^**	1058.3645	1058.3025	0.0620
23	86	[M − 9H_2_O − OH**^•^** − 6H]^6−1^**^•^**	157.1533	157.2115	−0.0582
24	84	[M − 2H_2_O − 3OH**^•^** + 3e − 3H]^6−^	173.0020	173.0518	−0.0498
25	51	[M − 1H**^•^** − H_2_O − 6H]^6−1^**^•^**	183.8332	183.8562	−0.0149

^a^The intensity relative to the base peak at *m*/*z* 1127.0719 in the spectrum with tallest peak set to 100%.

Along with the singly charged [M − *n*H_2_O − H]^−^ ions, six types of singly and multiply charged simple and distonic ion clusters and solvent adducts were detected within *m*/z 1000 and *m*/*z* 3000 (Figure 1 and Table 1): [M(n) − *n*H_2_O − xH]^x−^**,** [M(n) + H_2_O(m) − xH]^x−y•^, [M(n) + *m*H_2_O(m) − yH^•^ − xH]^x−y•^, [M(n) − *m*H_2_O − yH^•^ − xH]^x−y•^, [M(n) − *m*H_2_O − yHO^•^ + ye − xH]^(x+y)−^ and [M − *m*H_2_O − yHO^•^ − zH^•^ − xH]^x−(y+z)•^.

**Scheme 3:**
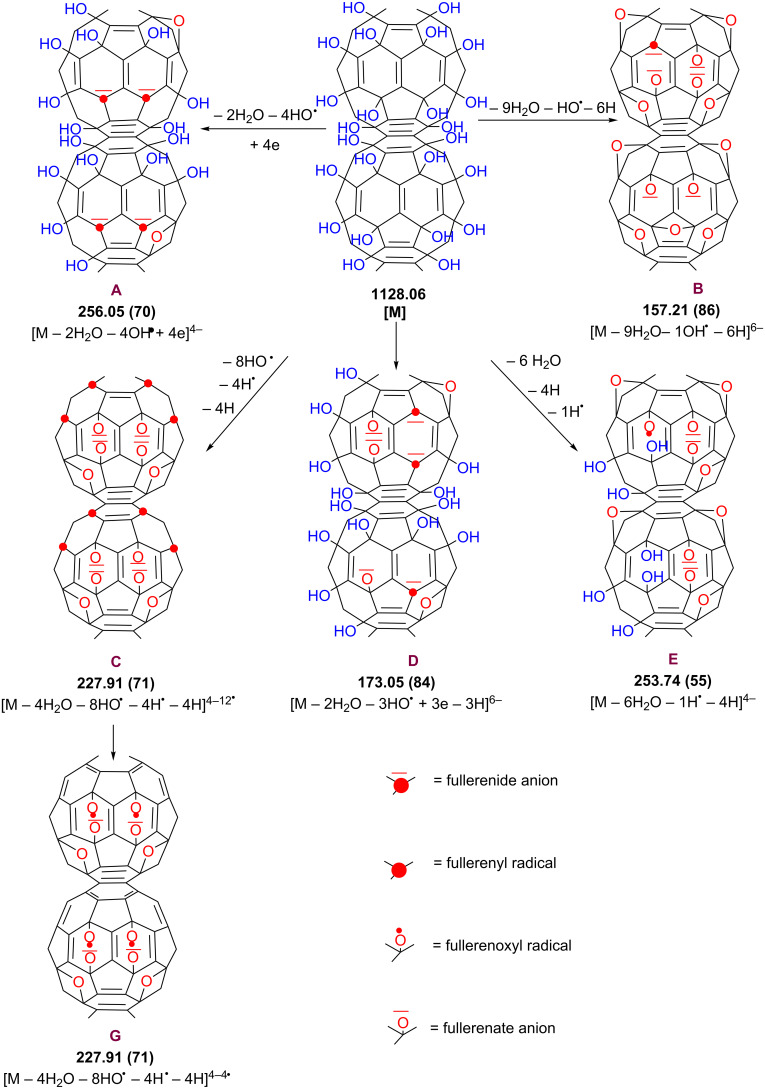
Proposed (−)ESI-MS ionization mechanisms for fullerenol C_60_(OH)_24_ in pure water.

Between *m*/*z* 100 and 600 (Table 1 and Scheme 3) the most intense peaks are designated to highly charged anions and distonic radical anions generated by ionization of the parent fullerenol C_60_(OH)_24_ as in the following examples: [M − 9H_2_O − HO^•^ + 5H]^6−1•^ at *m*/*z* 157 (Table 1, entry 23; **B** in Scheme 3); [M − 2H_2_O − 3HO^•^ + 3e − 3H]^6−^ at *m*/*z* 173 (Table 1, entry 24; **D** in Scheme 3); [M − 1H^•^ − 6H_2_O − 4H]^4−1•^ at *m*/*z* 253 (Table 1, entry 14; **E** in Scheme 3); and [M − 4H^•^ − 4H_2_O − 8HO^•^ − 4H]^4−4•^ at *m*/*z* 227 (Table 1**,** entry 13**; G** in Scheme 3). Above *m*/*z* 1500 the most intense peaks are assigned to the highly charged protonated and distonic deprotonated molecules containing 2–6 fullerenol and up to 66 H_2_O clusters.

Formation of these ions can be rationalized by the mechanisms shown in Scheme 1 and graphically depicted in Scheme 3. One can suppose that, in the electrospray source, M1-fullerenyl and M2-fullerenoxyl radicals are formed by neutral cleavage of C–OH and O–H bonds, respectively. As soon as fullerenyl radicals M1 containing a total of less than eight odd electrons with random distribution are generated, they readily capture free electrons present in the gas (due to cosmic rays or background radiation) to form fullerenide carbanion M3, while the fullerenoxyl radicals M2 do not capture free electrons, thus generating fullerenate anions M4. While only a few M1-fullerenyl radicals were detected as distonic anions [M(m) − *n*H_2_O − yHO^•^ − 3H]^3−y•^ at *m*/*z* 309, 335 and 781, fullerenoxyl radicals are much more resistant against free electrons and can be identified as abundant singly and multiply charged molecular anions containing up to 12 fullerenoxyl odd electrons.

A particular case is the abundant distonic anion [M − 4H_2_O − 8HO^•^ − 4H^•^ −4H]^4−12•^ at *m*/*z* 227 (entry 13 in Table 1, and **C** in Scheme 3), which appears to contain four fullerenoxyl radicals and eight fullerenyl radicals. In fact, the partial π-bond reconstruction by rehybridization and redistribution of odd fullerenyl electrons of intermediate **C** should account for the distonic anion **G**. The structure of the species [M − xH_2_O − yHO^•^ + ye − zH]^(z+y)−^ depicted as a high-range of relative abundance in the mass spectra, due to a loss of protons from fullerenyl-carbanion-containing species **M3,** could be assigned to fullerenide-fullerenate carbanion species **M6** (**B** and **D** in Scheme 3).

#### Fullerenol in ammonia aqueous solution

**Figure 2:**
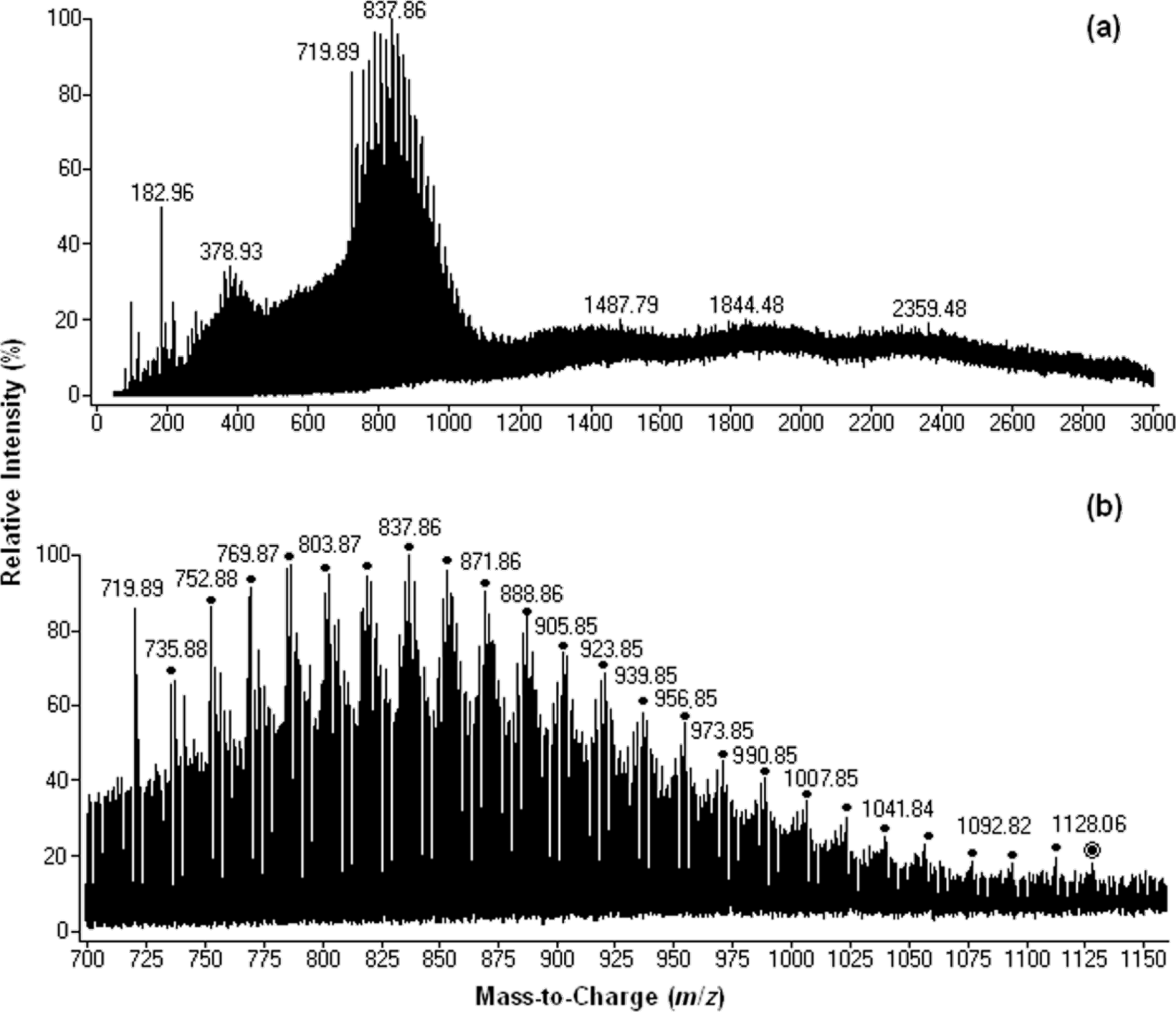
Negative-ion mass spectra of a 0.5 × 10^−5^ M aqueous solution of C_60_(OH)_24_ in ammonia solution: (a) full scan spectrum; (b) *m*/*z* ranges of 700–1200.

In order to enhance the ionization process of the fullerenol, small amounts of aqueous ammonia were used. Very abundant doubly charged ions with good intensity corresponding to dimeric species were observed for fullerenol C_60_(OH)_24_ in aqueous ammonia solution recorded at 4.5 kV capillary voltage and 400 V fragmentor voltage (Figure 2).

Consecutive losses of *m*/*z* 17 units are attributed to the neutral loss of *n*OH radicals (*n* = 2–48), followed by the rehybridization and redistribution of odd electrons of fullerenyl radical anion, accounting for the formation of a final molecular anion [2M − 48OH + 2e]^2−^ as observed at *m*/*z* 720 and depicted in Scheme 2**.** Taking into account the moderate electron affinity of fullerenols [32–33] and the fact that earlier extensive studies focused on pristine fullerene anion radicals [34–37] unquestionably confirmed their generation by electron transfer from various electron donors (aliphatic and aromatic amines especially) [38–39], one can postulate that, under ESI conditions, a distonic radical carbanion [(C_60_(OH)_24_**^−^**^•^)_2_]^2−•^ is first generated by electron transfer from H_2_N**^−^** produced by ammonia ionization and then undergoes consecutive OH radical losses and electron redistribution until the entire reconstruction of the electronic structure of pristine fullerene is achieved.

### Positive-ionization-mode mass spectra

**Figure 3:**
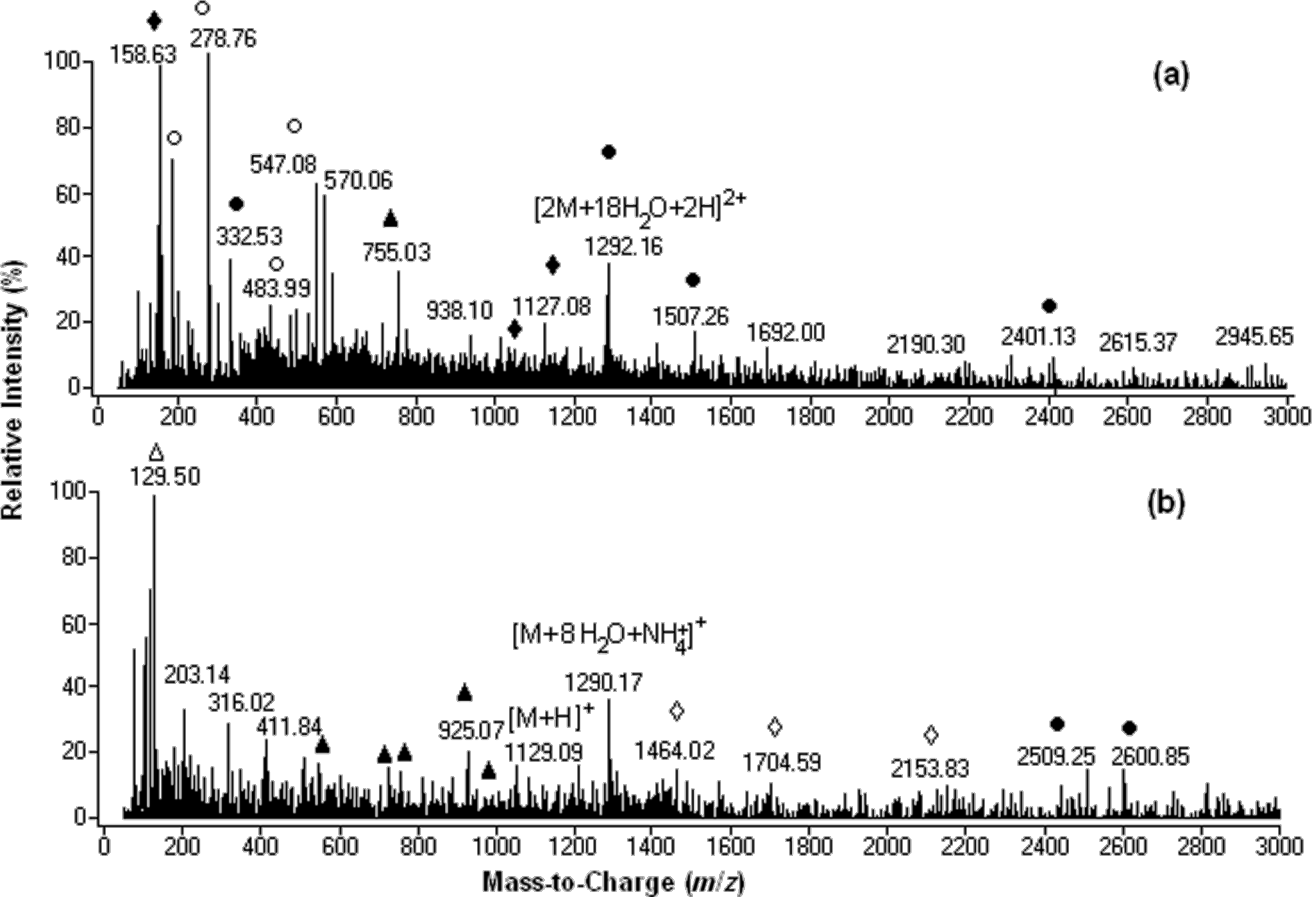
Positive ionization ESI mass spectrum of C_60_(OH)_24_ in (a) 3 × 10^−1^ M (b) 2 × 10^−2^ M aqueous ammonia solution. (●[M(n) + H_2_O(m) + xH]^x+^, ▲[M(n) − OH(m) + xH]^x+^, ♦[M(n) − H_2_O(m) + *n*NH_3_ + xH]^x+^, ○[M(n) − H_2_O(m) + xH]^x+^, ∆[M(n) − OH(m) + yNH_4_^+^ + xH]^(x+y)+^, ◊[M(n) + H_2_O(m) + *n*NH_3_ + xH]^x+^).

In Figure 3 are shown positive-ion-mode ESI mass spectra of C_60_(OH)_24_ with the addition of (a) 10 μL 3 × 10^−1^ M and (b) 10 μL 2 × 10^−2^ M aqueous ammonia solution. All of the observed ion species were obtained under 4.5 kV capillary voltage and 400 V fragmentor voltage.

With regard to molecular species and their stability, the positive-ion spectra of fullerenol in the presence of aqueous ammonia were significantly different from the negative-ion spectra. While the relative intensities of 2- to 6-protonated fullerenol with dilute 2 × 10^−2^ M ammonia aqueous solution were about threefold smaller than those observed under negative ionization conditions, the relative intensities recorded in the presence of 10 μL 3 × 10^−1^ M ammonia solution were about tenfold smaller than in the negative mode.

**Scheme 4:**
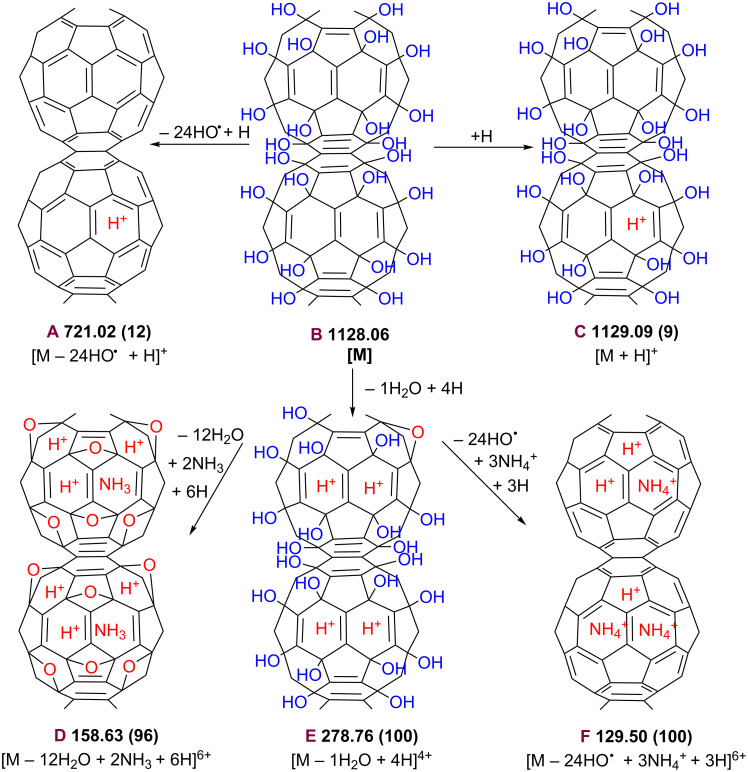
Proposed (+)ESI-MS ionization mechanisms for fullerenol C_60_(OH)_24_ in ammonia solution.

While the predominant base peak observed for C_60_(OH)_24_ in the negative mode was [M − H]^−^ those observed in the positive mode were [M − 12H_2_O + 2NH_3_ + 6H]^6+^ (96%) and [M − H_2_O + 4H]^4+^ (100%) (see Supporting Information File 1, Table S1, entries 9 and 7; **D** and **E** in Scheme 4) with 10 μL 2 × 10^−2^ M aqueous ammonia solution and [M − 24HO + 3NH_4_^+^ + 3H]^6+^ (100%) (see Supporting Information File 1, Table S1, entry 9; **F** in Scheme 4) with 10 μL 3 × 10^−1^ M aqueous ammonia solution.

The relative intensity of singly protonated fullerenol [M + H]^+^ (9%) at *m*/*z* 1129 was very low (Supporting Information File 1, entry 5 in Table S1 and **C** in Scheme 4). On the other hand, while under negative-ionization-mode conditions abundant [M(n) + H_2_O(m) − xH]^x−^ clustered ions with medium and high intensity are formed up to *n* = 6 and *m* = 66, only very few dimers, one tetramer and one pentamer with very low intensity were observed under positive-mode conditions.

## Conclusion

It is demonstrated here that electrospray mass spectrometry is a perfectly suitable method to study fullerenol C_60_(OH)_24_ in pure water and in the presence of aqueous ammonia solution in the negative and positive ionization modes, under the optimal capillary and fragmentor voltage. While the predominant base peak observed for C_60_(OH)_24_ in the negative-ionization mode was [M − H]^−^ at *m*/*z* 1127, those observed in the positive mode were multiply charged [M − H_2_O + 4H]^4−^ at *m*/*z* 279 (100%) and [M − 12H_2_O + 2NH_3_ + 6H]^6+^ at *m*/*z* 158 (96%).

We show that in the negative ionization mode, fullerenol C_60_(OH)_24_ readily lose not only H_2_O but also lose OH^•^ and H^•^ radicals giving rise to ion species that contain long-living fullerenoxyl radicals and/or short-living fullerenyl radicals, respectively. Affinity of short-lived fullerenyl radicals for free electrons to form carbanion-like fullerenyde charges on the C60 cage revealed an atypical pattern, characterized by neutral cleavage of C−OH bonds to form fullerenyl radicals, which can capture free electrons to generate negative charges, while fullerenoxyl radicals produced by O−H bond cleavage are much more stable against the free radicals.

In addition to simple bond cleavages, fullerenol ions containing at least eight fullerenyl radicals undergo an unanticipated gas-phase rearrangement that involves the partial (in the negative mode from pure aqueous media) or total (in the positive mode and negative mode from ammonia aqueous solutions) π-bond reconstruction of the C60 cage by rehybridization and redistribution of odd electrons of fullerenyl radicals to form C_60_ anions or cations, respectively.

## Experimental

### Materials

All chemicals and reagents used for fullerenol preparation and HPLC/ESI-MS analyses were commercially available (Sigma-Aldrich, St. Louis, MO, USA). The high purity C_60_(OH)_24_ sample was prepared as previously described [14–15] (see Supporting Information File 1). Freshly produced ultrapure water (TKA Lab TowerEDI 0.067–0.080 µS/cm) was used for sample preparation and as the mobile phase. The Tuning Mix solution for Q-TOF parameter optimization was purchased from Agilent Technologies.

### Sample preparation

A stock solution containing 0.5 × 10^−5^ M fullerenol C_60_(OH)_24_ was prepared by dissolution of dry fullerenol in ultrapure water, filtered and injected directly into the HPLC system. In order to prepare the samples with the ammonia medium, to each 1 mL of the 0.5 × 10^−5^ M fullerenol stock solution, 10 µL of 2 × 10^−2^ M or 3 × 10^−1^ M aqueous ammonia was added for two series of assays.

### HPLC separation conditions

HPLC separation was performed on a chromatographic system Agilent 1200 series separation module equipped with a binary pump, heated column compartment, autosampler and diode array detector. Separations were achieved using a new Agilent Zorbax SB-C18 reverse phase column (4.6 mm × 150 mm, 5 μm particle size) with a column temperature kept at 35 °C. Using a 20 μL injection volume, the fullerenol C_60_(OH)_24_ was monitored through UV detection at 280 nm for a total runtime of 30 minutes at a flow rate of 1 mL/min. Because the fullerenol C_60_(OH)_24_ is sparingly soluble in alcohols and acetonitrile, ultrapure water was used as the mobile phase for the HPLC separation and ESI-MS measurements, in negative- and positive-ionization mode. The mobile phase was filtered through a 0.45 μm filter and degassed for 30 minutes by sonication.

A typical chromatogram of the aqueous solution of C_60_(OH)_24_ (see Supporting Information File 1, Figure S1a) indicates a very small peak at approximately 1.5 min retention time, corresponding to contaminants from the sample, and a major peak at 2.6 min, corresponding to the analyte. The blank chromatogram (see Supporting Information File 1, Figure S1b) showed no peaks in the HPLC beside the solvent front. Hence, it is a clear evidence that the used column allows separation of C_60_(OH)_24_, and it is clear that fullerenol is essentially an individual pure compound.

### ESI-MS analysis

Mass spectrometry results were obtained using an Agilent 6520 Series Accurate-Mass Quadrupole Time-of-Flight (Q-TOF) LC/MS. The solutions were introduced into the electrospray ion source (ESI) after HPLC separation via a 4:1 splitter at a flow-rate of 0.2 mL/min. During the experiments, the following Q/TOF MS parameters were optimized: electrospray ionization (positive- and negative-ion mode), drying gas (N_2_) flow rate, drying-gas temperature, nebulizer pressure, capillary voltage, and fragmentor voltage. The mass scale was calibrated by the standard calibration procedure with compounds provided by the manufacturer. Data were collected and processed by using MassHunter workstation data acquisition software for the 6200/6500 series, version B.01.03. The calculated *m*/*z* values are based on the weight of the most abundant isotopes (monoisotopic mass).

### The key experimental observations

The first experiments studied the effect of ESI source parameters and mobile phase composition on the formation of fullerenol C_60_(OH)_24_ ions under negative and positive ionization mode. The fragmentor voltage and capillary voltage were the first parameters to be optimized because they have the greatest impact on the sensitivity and fragmentation. Consequently, the first objective was to find the optimum values for the fragmentor and capillary voltage that provides a strong molecular ion and a good relative abundance. The use of high voltages generates a greater signal, but this needs to be balanced with an increased background-to-noise ratio at the higher corona-current settings.

To optimize the conditions for obtaining maximum intensity, the capillary voltage was varied between 2.0 and 4.5 kV and the fragmentor voltage was varied between 100 and 400 V. Typical values for the other source parameters were: drying gas (N_2_) flow rate 8.0 L/min; drying-gas temperature 325 °C; nebulizer pressure 35 psig, skimmer 58 V and the octopol RF 750 V. The full-scan mass spectra of the investigated compounds were acquired in the range *m*/*z* 100–3000. The results are summarized in Figure S2 in Supporting Information File 1 showing the ESI-MS reference grid for C_60_(OH)_24_ as a function of fragmentor and capillary voltage in negative- and positive-ionization modes.

The experiments that were carried out at capillary voltage values of 2.0–4.0 kV and fragmentor voltage of 100–300 V (Supporting Information File 1, Figure S3a–c) did not affect any ionization and only peaks associated with unknown contaminants were observed at *m*/*z* 113, 294 and 420, detected in ultrapure water (Supporting Information File 1, Figure S1c) under negative-ionization-mode conditions. However, the fullerenol ionization takes place with good peak intensity distribution in the electrospray source at 4.5 kV capillary voltage and 400 V fragmentor voltage in negative-ionization mode (Figure 1 and Supporting Information File 1, Figure S3d) and at capillary voltage range of 3.0–4.5 eV in positive-ionization mode. Although a capillary voltage of 4.5 kV was recently reported to effect positive ionization of the MER commercial fullerenol C_60_(OH)_16_(ONa)_8_ [23] the reported results based only on a full scan spectrum of MER fullerenol sample were completely different from the current reported results.

## Supporting Information

File 1 Additional material.
