# Supplementary material for: A study on electrospray mass spectrometry of fullerenol C60(OH)24
Source: Beilstein J Org Chem. 2013 Jul 2;9:1285–95. doi: 10.3762/bjoc.9.145 (PMC3701416; doi:10.3762/bjoc.9.145)
Supplement: File 1 — Additional material. [file Beilstein_J_Org_Chem-09-1285-s001.pdf]

Supporting Information

for

A study on electrospray mass spectrometry of fullerenol $C_{60}(OH)_{24}$

Mihaela Silion[1], Andrei Dascalu[1*], Mariana Pinteala[1*], Bogdan C. Simionescu[1,2] and Cezar Ungurenasu[1]

Address: [1]Petru Poni Institute of Macromolecular Chemistry, Centre of Advanced Research in Bionanoconjugates and Biopolymers, Aleea Grigore Ghica Voda 41A, Iasi 700487, Romania and [2]Department of Natural and Synthetic Polymers, "Gheorghe Asachi" Technical University of Iasi, 700050 Iasi, Romania

Email: Andrei Dascalu - idascalu@icmpp.ro; Mariana Pinteala - pinteala@icmpp.ro

*Corresponding author

Additional Material

Synthesis of Fullerenol $C_{60}OH_{24}$ [1]

All experiments were performed with Schlenk techniques under argon and protected from light. According to the literature, before the synthesis of the polyhydroxylated fullerene,

bromofullerene $C_{60}Br_{24}$ was synthesized [2]. In the synthesis of the $C_{60}(OH)_{24}$, to a sonicated (40 W, 15 min) suspension of $C_{60}Br_{24}$ (200 mg, 0.075 mmol) in deaerated water (100 mL), fresh KOH (200 mg, 3.57 mmol) was added under argon protection and stirred for 10 days at room temperature. After the reaction was completed, the resulting dark-brown solution was passed to a centrifuge at 4000 rpm for 30 min and the supernatant was brought to dryness in a rotavapor apparatus at 40 °C. The dark-brown residue was dissolved in 50 mL of deionized water, stirred with ion exchange resin AMBERJET™ 1200[H] (Rohm and Haas Company, Philadelphia, PA, USA) (20 mL) for 8 h, and subjected to dialysis (Spectra/Por® 1000 D; Spectrum Laboratories, Rancho Dominguez, CA, USA) for four days with ultrapure water. Finally, the dialyzed solution was brought to dryness in a rotavapor apparatus at 60 °C and dried at 80 °C and 10^{-4} Torr for 24 h. The fullerenol thus obtained contained 24 hydroxy groups as characterized by elemental analysis, Fourier transform infrared (FTIR) spectroscopy, MALDI-MS and XPS spectroscopic measurements.

Elemental analysis: Calculated for $C_{60}H_{24}O_{24}$: C, 63.82; H, 2.12. Found: C, 63.66; H, 1.98; FTIR (KBr): v max: 1430 (–OH), 1605 (C =C), 1095, 1046 (C-OH), 1018, 994, 877, 570, 530 cm^{-1}; MW (MALDI-TOF): Calculated for $C_{60}H_{24}O_{24}$: 1128.0570. Found: 1128.0610; XPS analysis: C1s components: % C=C (284.6 eV) 59.77 (clcd, 60); % C–OH: (285.8 eV) 39.76 (clcd. 40); O/C: 0.42 (clcd. 0.40)

References

[1] Pinteala, M.; Dascalu, A.; Ungurenasu, C. Int. J. Nanomedicine **2009**, 4, 193.

[2] Troshin, P.A.; Astakova, A.S.; Lyubovskaya, R.N. Fullerenes Nanotubes Carbon Nanostruct. **2005**,13, 1–13.

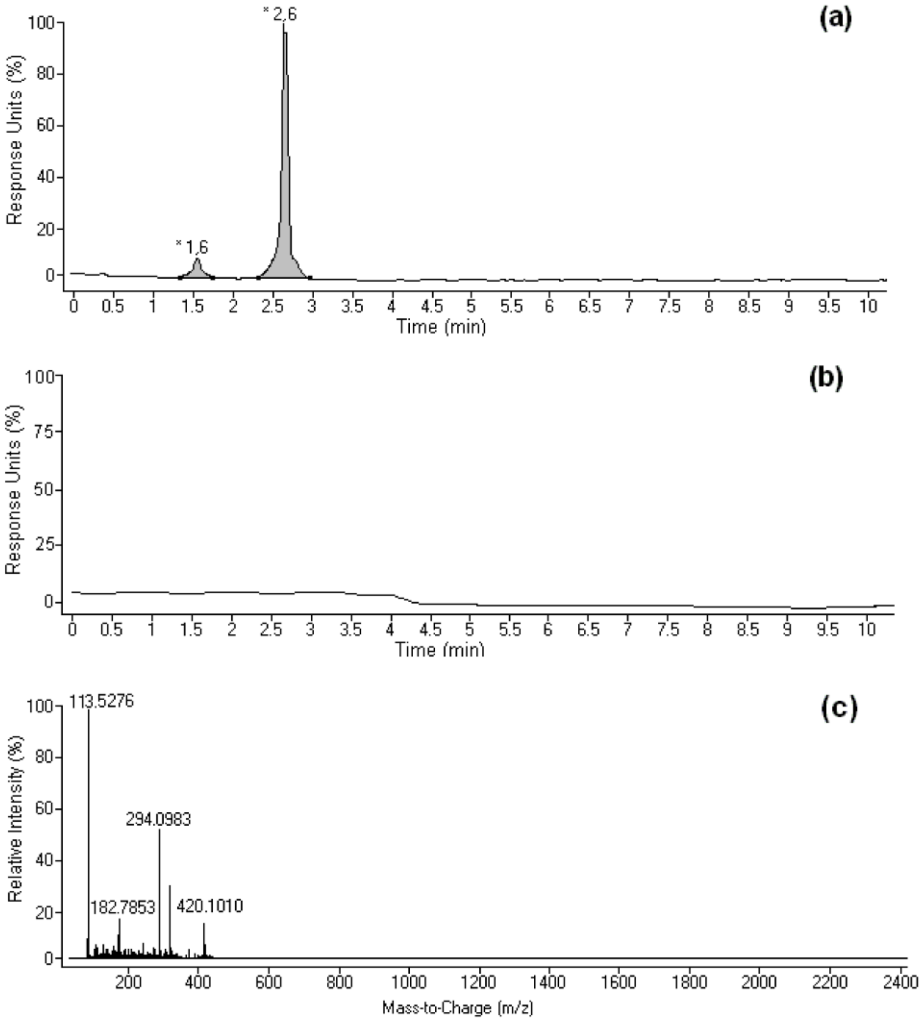

Figure S1: Typical chromatogram for the HPLC-ESI-MS analysis of $C_{60}(OH)_{24}$ on a Zorbax SB-C18 reverse-phase column and ultrapure water mobile phase (a), blank chromatogram (b) and ESI-MS spectrum of blank mobile phase (c).

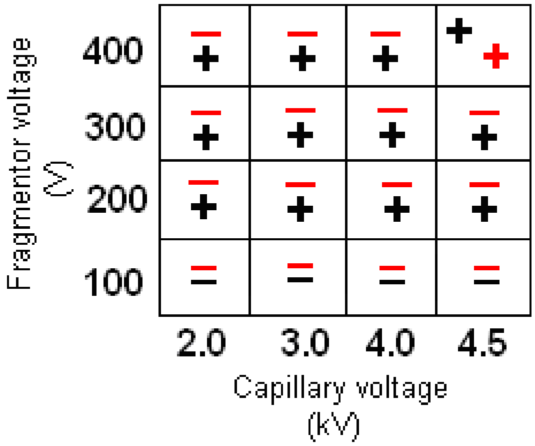

Figure S2: An ESI-MS reference grid for $C_{60}(OH)_{24}$ as a function of fragmentor (black) and capillary voltage (red) under negative and positive ionization mode.

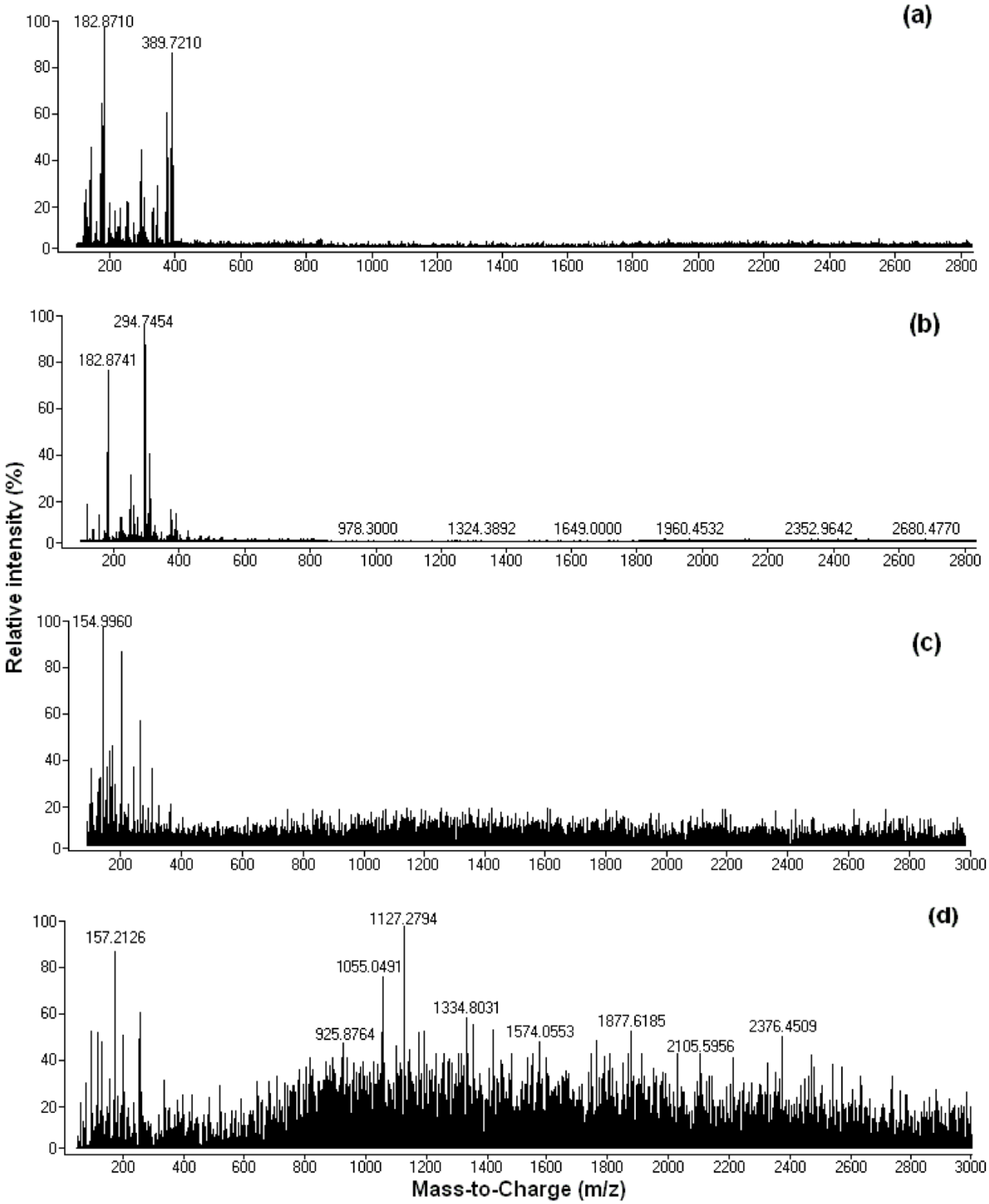

Figure S3: Typical ESI-MS spectra of $C_{60}(OH)_{24}$ (0.5×10^{-5} M) in pure water media at 4.5 kV capillary voltage and variable fragmentor voltage: (a) 100 V; (b) 200 V; (c) 300 V; (d) 400 V.

Table S1: Summary of the dominant ESI-MS positive ionic species of fullerenol $C_{60}(OH)_{24}$ in ammonia solution (relative abundance, assignments and *m*/*z* values) at 4.5 kV capillary voltage and 400 V fragmentor voltage

Entry	Relative intensity (%)[a]	Detected quasimolecular ion	*m*/*z*		
			Calculated	Found	Deviation
**3×10^{-1}M ammonia**					
1	36	$[M - 22OH + H]^{+}$	755.0132	755.0289	−0.0157
2	15	$[M + 21H_2O + H]^{+}$	1507.2952	1507.2640	0.0312
3	10	$[2M + 8H_2O + H]^{+}$	2401.2222	2401.1333	0.0889
4	25	$[M - 9H_2O + 2H]^{2+}$	483.9925	483.9903	0.0022
5	62	$[M - 2H_2O + 2H]^{2+}$	547.0296	547.0846	−0.055
6	38	$[2M + 18H_2O + 2H]^{2+}$	1292.1680	1292.1605	0.0075
7	100	$[M - H_2O + 4H]^{4+}$	278.7733	278.7631	0.0102
8	39	$[M + 11H_2O + 4H]^{4+}$	332.5531	332.5303	0.0228
9	96	$[M - 12H_2O + 2NH_3 + 6H]^{6+}$	158.6729	158.6288	0.0441
10	63	$[M - 1H_2O + 6H]^{6+}$	186.0168	185.9954	0.0214
**2×10^{-2} M ammonia**					
1	12	$[M - 24OH + H]^{+}$	721.0078	721.0157	−0.0079
2	10	$[M - 23OH + H]^{+}$	738.0105	738.0612	−0.0507
3	21	$[M - 12OH + H]^{+}$	925.0402	925.0687	−0.0285
4	14	$[M - 3OH + H]^{+}$	1078.0645	1078.0263	0.0382
5	9	$[M + H]^{+}$	1129.0726	1129.0886	−0.016
6	35	$[M + 8H_2O + NH_4^{+}]^{+}$	1290.1840	1290.1696	0.0144
7	15	$[2M + 14H_2O + H]^{+}$	2509.2858	2509.2474	0.0384
8	18	$[2M - 4OH + 4H]^{4+}$	548.0375	548.0293	0.0082
9	100	$[M - 24OH + 3NH_4^{+} + 3H]^{6+}$	129.5211	129.5004	0.0207
10	35	$[M + 5NH_4^{+} + 1H]^{6+}$	203.2074	203.1401	0.0673

[a]The intensity relative to the base peak at *m*/*z* 278.7631 and 129.5004 in the spectrum with tallest peak set to 100%.
